# Seedborne mycoflora of faba bean (*Vicia fabae* L.) and evaluation of plant extract and *Trichoderma* species against mycelium growth of selected fungi

**DOI:** 10.1016/j.heliyon.2023.e17291

**Published:** 2023-06-15

**Authors:** Amsalu Neme, Ararsa Leta, Amin Mohammed Yones, Muhidin Tahir

**Affiliations:** a Department of Plant Science, College of Agriculture and Veterinary Science, Ambo University, Ethiopia; b Department of Horticultural Sciences, College of Agriculture, Oda Bultum University, P.O. Box 226, Chiro, Ethiopia; c Department of Biology, College of Natural and Computational Sciences, Oda Bultum University, P.O. Box 226, Chiro, Ethiopia

**Keywords:** Faba bean, Mycelial growth, Mycoflora, Plant extract, *Trichoderma* spp.

## Abstract

Fungal diseases are among the biotic factors limiting the production of faba bean in Ethiopia. The objective of this study was to isolate and identify seedborne mycoflora associated with faba bean seed samples, determine their effects on seed germination and disease transmission, and evaluate the antimicrobial activities of seven plant extracts and four *Trichoderma* spp. against the pathogen isolated from the seed. Fifty seed samples were collected from different farmers’ saved seeds of five major faba bean-producing varieties of the Ambo district and were tested by agar plate methods as recommended by the International Seed Testing Association (ISTA). A total of 7 fungal species belonging to 6 genera, viz. *Fusarium oxysporum* (Schlechlendahl), *Fusarium solani* (Mart.) Sacc, *Aspergillus* spp. *Penicillium* spp. *Botrytis* spp. *Rhizoctonia solani* (Kühn) and *Alternaria* spp. were isolated and identified. Among these, *Fusarium* spp., *Aspergillus* spp, and *Penicillium* spp. were the most predominant fungi in all seed samples. Seed-to-seedling transmission test results confirmed that *F. oxysporum*, *F. solani and R. solani* were major causal pathogens that caused root rot and damping-off disease in faba beans and were transmitted from seeds to seedlings. A higher germination rate was observed in Golja-GF_2_ (97%), and a lower germination rate was observed in Kure Gatira-KF_8_ (81%). A study on in vitro evaluation of plant extract and *Trichoderma* spp. against *F. oxysporum*, *F.* solani and *R. solani* revealed that plant extracts at 5%, 10% and 20% concentrations significantly inhibited the mycelial growth of all tested fungi. Inhibitory effects on the three tested fungi (*R. solani, F. solani and F. oxysporum*) were recorded on *T. longibrachiatum* (87.91%), *T. atroviride* (86.87%), *Trichoderma virens* (86.16%) and *T. harzianum* (85.45%). The inhibitory effect of the aqueous plant extracts on mycelial growth increased with an increase in concentration, and the hot water extracts showed higher effects compared to the cold water extract in all tested fungi. This study showed that the highest inhibitory effect of *Allium sativum* L. extracted at a 20% concentration against mycelial growth inhibition of the three test fungi *(F. oxysporum, R. solani* and *F. solani*) was 84.60%, 83.61% and 83.47%, respectively. However, *Nicandra physalodes* (L.) Gaertn.) extracts at the same concentration showed the lowest inhibitory effects on the three tested fungi (74.94%, 73.94% and 73.24%).

## 1 Introduction

Faba bean (*Vicia fabae* L.) is one of the most important food legumes in the world due to its high nutritive value both in terms of energy and protein contents containing 24–30% [[1], [2], [3]], and it is also an excellent nitrogen fixer [4]. It ranks fourth in the world after garden peas, chickpeas and lentils. Its production worldwide is concentrated in nine major agroecological regions, namely, northern Europe, the Mediterranean, the Nile valley, Ethiopia, Central Asia, East Asia, Oceana, Latin America, and North America [5].

It is an important high-land pulse crop of Ethiopia, which covers 520,519 ha of cultivated land with an annual production of 930,633 tons [6]. The crop accounts for the largest share of the area under pulse production in Ethiopia. The growing importance of this crop as an export in Ethiopia has led to a renewed interest by farmers to increase the area under production [7,8].

Despite its wide cultivation and huge importance, the average yield of *Vicia fabae* L. is quite low in Ethiopia. The average national yield is approximately 2.1 t ha^−1^ [9], which is very low compared to the average yield of 3.7 t ha^−1^ in major producer countries [10]. The productivity of this crop is far below its potential because of several biotic and abiotic constraints [11]. According to Sahile et al. [8], diseases are the most important biotic factors limiting the production of faba bean in Ethiopia. It is attacked by more than 100 pathogens [12]. More than 17 pathogens have been reported in Ethiopia [13]; among these, fungi are the largest and perhaps the most important groups affecting all parts of these plants at all growth stages [14].

Fungal diseases such as chocolate spot (*Botrytis fabae*), rust (*Uromyces fabae*), black root rot (*Fusarium solani*), and foot rot (*Fusarium avenaceum*) are among the fungal pathogens that contribute to the low productivity of the faba bean [15].

Seed dressings are used to eliminate most surface infestations of seeds but have relatively little effect on internally borne organisms [16]. An urgent need for alternatives to fungicides for the control of seedborne fungi is important in recent years; much attention has been given to nonchemical systems for seed treatment to protect them against seedborne pathogens. Plant extracts have played a significant role in the inhibition of seedborne pathogens and the improvement of seed quality and field emergence of seeds. Many authors have reported the effective and safe use of plant extracts for controlling seedborne fungi [[17], [18], [19], [20]].

In the current study, the effects of seedborne fungi on seed germination, seed-to-seedling transmission and their management are important for the improvement of crop production and productivity. Therefore, this study was initiated with the following specific objectives: to assess the fungal pathogens associated with farmers' saved seeds in the study areas; to determine the level of seed infection by fungal pathogens associated with farmers' saved seeds in the study area; to determine the effect of seed infection on seed germination and disease transmission; and to evaluate the efficacy of some plant extracts and *Trichoderma* spp. against fungi associated with faba bean seeds.

## 2 Materials and methods

### 2.1 Description of the study areas

**Fig. 1:**
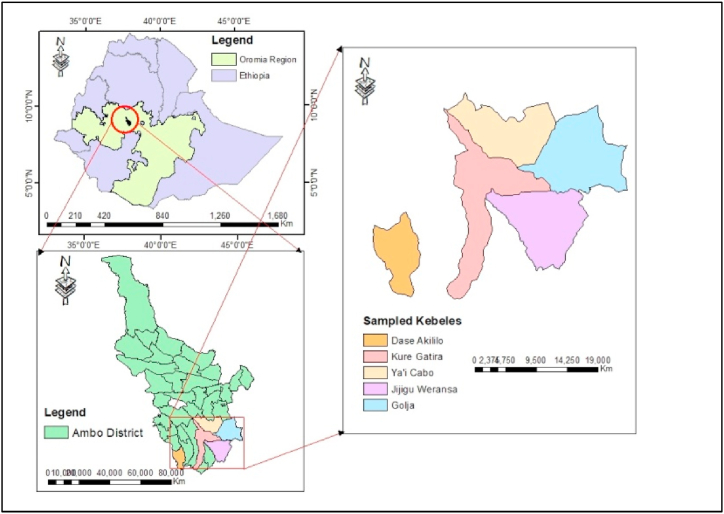
Map of the study area.

The seed samples were collected from the major faba bean growing farmers’ association of Ambo district, and the laboratory study was conducted at Ambo University College of Agriculture and Veterinary Science. Ambo is located 120 km west of Addis Ababa at 8° 98′ south latitude and 37° 83′ E 37° 83′ north longitude (Fig. 1). It has a total geographical area of 83,598.69 sq. km., with elevations ranging from 1380 to 3300 m a.s.l. The annual rainfall ranges from 900 to 1100 mm, and the temperature ranges from 10 to 27 °C, with an average of 18 °C (National Meteorological Service, unpublished data of 2018).

### 2.2 Seed sample collection and isolation of fungi

A total of 50 seed samples were collected in polythene envelopes from different farmers’ saved seeds of five major faba bean-producing farmer associations, namely, Golja, Jijigu Weransa, Ya’i Cabo, Kure Gatira, and Dase Akililo, in Ambo district with consults of the district agricultural office. The purposive sampling method was used for selecting the farmers’ association, and seed samples were taken according to the [21] sampling procedure. Each sample was enclosed in paper bags with proper labelling, taken to the laboratory, and then kept in a refrigerator at 4 °C for 24 h until the beginning of the identification tests.

Four hundred seeds were randomly taken from each seed sample for mycoflora study. Four replicates of 100 seeds per sample were surface sterilized using 0.5% sodium hypochlorite (NaOCl) solution for 10 min with constant agitation. Seeds were rinsed three times in sterile distilled water for a minute, placed on sterile Whatman filter paper to dry and inoculated with 10 seeds per plate aseptically in (90 mm diameter) Petri dishes containing PDA using a sterile pair of forceps.

Ten Petri dishes were plated with 100 seeds to represent the replicates of treatment, and these were arranged in a completely randomized design. All Petri dishes were incubated at 25 ± 2 °C for 7 days under refrigeration cycles of 12 h near ultraviolet (UV) light and 12 h of darkness for sporulation of fungi. After incubation, the growth characteristics, as well as the percentage of infection, were recorded. The isolated colony culture of fungi was maintained on potato dextrose agar (PDA) medium. The fungi were identified based on their morphological features, as used by Booth [22] and Barnett and Hunter [23].

### 2.3 Determination of seeds infected with fungi

The percentage of seeds with fungal infection was determined by counting the infected seeds and dividing by the total number of seeds and expressing them as a percentage. Thus,

(1) $$Percent\;of\;seeds\;infected=\frac{No\;seed\;infected\;with\;fungi}{Total\;no\;of\;seeds\;per\;plate}\times 100$$

Colonies obtained from each infected seed were subjected to cultural and microscopic morphological characterization for identification. The occurrence of fungi was determined by counting the number of times each fungus occurred divided by the total number of fungi and expressed as a percentage. Thus,

(2) $$Percentage\;occurrence\;of\;fungi=\frac{No\;of\;times\;each\;fungus\;occurred}{Total\;no\;of\;fungi\;per\;plate}\times 100$$

The identification of the fungi was based on cultural characteristics, mainly on the growth patterns and pigmentation. Further microscopic examinations were carried out for mycelial and conidia structures based on the user identification manual of illustrated genera of fungi [23,24]. The morphological characteristics were determined by taking a small amount of mycelium from ten-day-old pure culture plates using a sterile needle and transferring onto a cleaned glass slide. The culture was taken from five different positions of the culture on the plate, four from the adjacent side and one from the middle. The mycelium was stained with 0.1% lactophenol cotton blue and observed under a compound microscope.

### 2.4 Effect of seed borne myco-flora on seed germination

To examine the effect of seedborne fungi on seed germination and seed-to-seedling transmission, seedling bioassays were carried out in a growth chamber. The sand used in the studies was sterilized in an autoclave at 121 °C and a pressure of 15 lb for 1 h, after which it was ready for use and allowed to dry. One hundred seeds were randomly taken from each sample and were surface sterilized by soaking in 0.5% sodium hypochlorite (NaOCl) solution for 10 min with constant agitation followed by rinsing in three changes of sterile distilled water for a minute and then dried between two layers of sterilized Whatman filter paper. One hundred treated seeds were placed in a tray containing sterilized sand aseptically by using a sterilized pair of forceps at equidistance to avoid cross-contamination and then placed in a growth chamber at 28 ± 2 °C in alternating cycles of 12 h of darkness and 12 h of light. The seeds were kept moistened by adding approximately 3 mL of sterilized distilled water every two days throughout the incubation period. The effect of the seedborne fungi on the germination of faba bean seed samples was determined after 7 days of incubation. Seed germination was assessed by counting the seeds with seed leaves and dividing them by the total number of seeds in the Petri plates expressed as a percentage.

(3) $$Germination\;percentage=\frac{Number\;of\;seeds\;germinated\times 100}{Total\;no\;of\;seeds\;per\;plate}$$

The incidence of the fungi in coleoptiles and root tissues of seedlings was determined on the 7th, 14th and 21st day after plating in sterilized sand by pulling out the seeds/seedlings showing disease symptoms. Finally, the seedling infection percentages per plate were calculated [25].

(4) $$Seedling\;infection\;percentages=\frac{No.\;of\;seedlings\;affected\;by\;a\;pathogen\times 100}{Total\;number\;of\;seeds\;sown}$$

In addition, the progress of the disease and symptoms developed were monitored in the plated plants. The transmission efficiency (TE) of fungi from seeds to seedlings was estimated from the incidence data with the following formula:

(5) $$Transmission\;efficiency\;of\;fungi\;from\;seeds\;to\;seedling=\frac{C\times 100}{S}$$

where C is the infection percentage of seedlings by fungi in the transmission study and S is the seed infection percentage of the fungi during the laboratory assay on agar. In vitro antimicrobial assays of the plant extracts and bioagents were performed in the plant pathology laboratory of Ambo University College of Agriculture and Veterinary Science.

### 2.5 Evaluation of plant extract

The fresh aerial and underground plant parts of Garlic clove (*A. sativum* L.), Kombolcha leaf (*Maytenus senegalensis* Loes.), Argissa (*Aloe vera* (L.) Burm.f), Abshoo leaf (*N. physalodes* (L.) Gaertn.), tabaco leaf (*Nicotiana tabacum* L.), Turmeric rhzome (*Curcuma longa* L.) and endod seeds (*Phytolacca dodecandra* L’Hér.) used in this study were collected in December 2019. *A. sativum* L. and *M. senegalensis* Loes. were collected from the Ambo district Amaro areas. *A. vera* (L.) Burm.f, *N. physalodes* (L.) Gaertn. and *N. tabacum* were collected from the Toke Kutaye district Guder areas. *C. longa* L. was collected from the west Wellaga zone (Gimbi), and *P. dodecandra* was collected from the Jibat state forest, west Shewa. The samples were separated into their selected parts (leaf, clove and seed), washed thoroughly under tap water followed by sterilized distilled water, cut into a smaller size of approximately 1–3 cm long, air dried under shade at room temperature for 1–2 weeks, pounded using a sterile mortar and pistil into a fine powder and kept in the refrigerator at 4 °C until use [26,27].

The crude plant extract was obtained by socking/infusing 50 g of each air-dried powdered plant material in 500 ml of hot and cold distilled water separately (1:10 w/v) in a 1000 ml conical flask. For garlic and *A. vera* (L.) Burm.f, 50 g of garlic paste and 50 g of *A. vera* (L.) Burm.f were placed into a 1000 ml conical flask and then filled with hot and cold distilled water separately up to 500 ml to give 1:10 w/v. The flasks were kept on a rotary shaker for 30 min, and then extraction took place under cold conditions for 24 h [28]. Plant extracts were filtered through double layers of muslin cloth followed by Whatman filter paper. Finally, the aqueous extract at a concentration of 10% was used as an original concentration in the antifungal activity test and stored in an airtight bottle in a refrigerator at 4 °C for further usage [29].

Five plant parts (leaf, clove, gel, rhizome, and seed) were evaluated against mycelial growth of seedborne test fungi *F. solani*, *F. oxysporum* and *R. solani* by using the poisoned food technique at 5, 10 and 20% concentrations for which 5, 10 and 20 ml of crude extract stock solution were mixed with 95, 90 and 80 ml of sterilized molten PDA media. The medium was thoroughly shaken for uniform mixing of leaf extract, and 20 ml of agar media was poured into sterile Petri plates and allowed to solidify. Five millimeter agar disks of test fungi were cut from 7-day-old culture plates by using a sterile cork borer and placed in the centre of the Petri plate containing different concentrations of plant extract. The experiment was conducted in a complete randomized design (CRD), with 7 treatments and 3 replications for each test pathogen. The Petri plates containing only PDA medium without plant extracts served as a control. All inoculated Petri plates were incubated at 25 ± 2 °C for seven days, and the data on the mycelial growth of the fungus were recorded from 24 h of inoculation to 7 days of inoculation. The percentage inhibition of mycelial growth was calculated as per the formula given by [30].

(6) $$Percent\;inhibition\;of\;test\;fungi=\frac{\left(C-T\right)}{C}\times 100$$

where C is the average increase in mycelial growth in the control plate and T is the average increase in mycelial growth in the treatment plate.

### 2.6 Evaluation of bioagents under in vitro conditions

Purified cultures of antagonistic *Trichoderma* isolates *T. harzianum, T. longibrachiatum, T. atroviride*, and *T. virens* used in this study were obtained from the Plant Pathology Department of Ambo University College of Agriculture and Veterinary Sciences. Stock cultures of *Trichoderma* spp. contained pertinent information regarding how they were isolated from the natural environment and maintained on Petri dishes containing PDA medium in a refrigerator at 4 °C for subsequent use.

Stock cultures of four *Trichoderma* species were recultured in sucrose peptone broth (SPB) for multiplication, as recommended by Kumar et al. [31]. The conidia (colony-forming unit, cfu) suspension of each species was prepared in sterile distilled water from a 7-day-old culture on PDA [32]. The fungal inoculum was harvested by flooding the culture with sterile distilled water (SDW) and then rubbing the culture surface with a sterile glass rod. The fungal propagule concentration in each suspension was determined by counting using a hemocytometer slide, adjusted to 10^8^ cfu/ml and used in a dual culture test following Kumar et al. [31].

The antagonistic effect of *Trichoderma* species against *F. oxysporum, F. solani* and *R. solani* isolates was evaluated by using the dual culture technique [33]. All the isolate test fungi and *Trichoderma* spp. were grown separately on sterilized standard PDA at 25 ± 2 °C in an incubator for 5 days to obtain juvenile colonies for the studies of antagonism. After an incubation period of 5 days, 5-mm diameter mycelial plugs of each isolated test fungus were cut by using a sterile cork borer and placed at the periphery of culture plates amended with tetracycline (0.1 g/L) by leaving 2 cm away from the edge side of the Petri dish, and on the same day, antagonist *Trichoderma* species were placed with equal distance on the opposite side of the same previous Petri dishes. For each treatment, three replications were used. The control plate was maintained by inoculating the medium with the pathogen only. The plates were incubated at 25 ± 2 °C in an incubator [34]. The growth of the pathogen in both the test and control experiments was recorded. The percent inhibition of the isolated test fungi was calculated by using the following formula of Vincent [30]:

(7) $$Percent\;inhibition\;of\;test\;fungi=\frac{\left(R1-R2\right)}{R1}\times 100$$

where R1: the radial colony growth of test fungi in the control plate (mm); R2: the radial colony growth of test fungi in the dual culture plate (mm) and the width of the zone of inhibition (ZI) was measured as the smallest distance between the colonies in the dual culture plate [[35], [36], [37]].

### 2.7 Data analysis

The obtained data were statistically analysed according to analysis of variance (ANOVA); Duncan's multiple range test was used for mean separation using SAS software for Windows version 9.2.

## 3 Results

### 3.1 Isolated fungi and determination of infected seeds

Seedborne mycoflora of faba bean was tested by using the agar plate method as recommended by ISTA. Of the 50 farmers’ saved seed samples collected from the five major faba bean-producing farmer associations in Ambo District, a total of 7 fungal species, *F. oxysporum, F. solani, Aspergillus* spp*., Botrytis* spp., *Penicillium* spp., *R. solani* and *Alternaria* spp. belonging to 6 fungal genera were isolated (Table 1).

**Table 1:** Seed germination and percent seed infection of different faba bean seed samples in the Ambo district.

Location	Seed sample code	Germination %	Percent seed infection (%)							
			*F. oxysporum*	*F. solani*	*Aspergillus* spp.	*Penicillium* spp.	*Botrytis* spp.	*Rhizoctonia solani*	*Alternaria* spp.	Total
Golja	GF_1_	93	62	23	24	0	0	22	36	41.75
	GF_2_	96	14	16	29	0	51	15	0	31.25
	GF_3_	100	56	50	0	29	15	35	20	51.25
	GF_4_	96	68	20	0	46	0	20	58	53.00
	GF_5_	93	43	50	60	0	0	1	0	38.50
	GF_6_	98	62	61	32	0	20	2	0	44.25
	GF_7_	94	42	52	20	53	58	1	50	69.00
	GF_8_	100	43	62	21	0	57	45	27	63.75
	GF_9_	100	17	60	27	0	60	23	20	51.75
	GF_10_	100	62	53	0	39	43	38	52	71.75
Jijigu Weransa	JF_1_	75	48	40	0	30	58	54	48	69.50
	JF_2_	100	55	26	0	52	60	2	0	48.75
	JF_3_	84	39	26	40	0	38	56	54	63.25
	JF_4_	100	52	18	38	46	51	1	0	51.50
	JF_5_	84	45	20	30	29	30	2	20	44.00
	JF_6_	100	62	39	30	45	59	2	67	76.00
	JF_7_	70	67	30	30	20	0	1	26	43.50
	JF_8_	100	38	32	60	60	60	1	50	75.25
	JF_9_	100	32	44	71	40	51	40	62	85.00
	JF_10_	75	54	53	0	55	53	1	67	70.75
Ya’i Cabo	YF_1_	78	72	45	39	60	30	2	39	71.75
	YF_2_	96	55	55	45	30	44	2	0	57.75
	YF_3_	94	46	57	46	0	0	62	0	52.75
	YF_4_	100	52	60	30	51	0	3	0	49.00
	YF_5_	95	24	52	56	30	57	1	58	69.50
	YF_6_	76	52	45	62	68	10	20	58	78.75
	YF_7_	78	72	56	0	73	0	60	20	70.25
	YF_8_	75	42	46	38	0	65	58	54	75.75
	YF_9_	94	30	52	68	36	0	20	0	51.50
	YF_10_	64	69	52	74	48	36	66	0	86.25
Kure Gatira	KF_1_	100	72	60	74	60	0	2	70	84.50
	KF_2_	68	31	51	57	0	0	56	0	48.75
	KF_3_	75	74	68	0	56	0	3	0	50.25
	KF_4_	67	73	60	50	63	16	54	0	79.00
	KF_5_	83	72	63	74	0	68	3	0	70.00
	KF_6_	76	55	70	72	53	0	30	0	70.00
	KF_7_	78	56	72	55	44	0	50	29	76.50
	KF_8_	89	76	59	39	48	29	55	43	87.25
	KF_9_	78	52	45	56	20	39	2	72	71.50
	KF_10_	96	47	56	20	43	0	65	0	57.75
Dase Akililoo	DF_1_	91	45	71	62	68	19	16	0	70.25
	DF_2_	88	49	61	42	0	0	50	0	50.50
	DF3	87	40	46	49	57	0	30	49	67.75
	DF_4_	85	48	31	60	30	60	39	0	67.00
	DF_5_	97	65	48	30	47	0	40	0	57.50
	DF_6_	79	47	65	48	15	57	1	58	72.75
	DF_7_	75	64	65	55	10	0	3	0	49.25
	DF_8_	100	51	70	46	68	0	17	58	77.50
	DF_9_	74	50	59	27	22	0	36	3	49.25
	DF_10_	84	42	50	67	59	0	70	0	72.00
Mean		87.56	20.6	19.65	15.57	13.58	10.31	10.19	10.11	62.73

GF_1-10_ = Sample collected from Golja, JF_1-10_ = Sample collected from Jijigu Weransa, YF_1-10_ = Sample collected from Ya’i Cabo, KF_1-10_ = Sample collected from Kure Gatira, DF_1-10_ = Sample collected from Dase Akililu.

Among the isolated fungi, *F. oxysporum, F. solani* and *Aspergillus* spp*.* had higher incidences (12.92%, 12.33% and 9.77%, respectively), followed by *Penicillium* (8.52%) and *Botrytis* spp. (6.47%), and lower incidences were *R. solani* (6.39%) and *Alternaria* spp. (6.34%). Samples from Kure Gatira kebele showed higher infection (88.25%), whereas samples collected from Golja kebele showed lower infection (31.25%) (Table 1).

### 3.2 Identification of fungi and frequency of occurrence

In this study, visual and microscopic observations were used to characterize the selected colony cultures. Details of the hyphal and spore characteristics of the fungi are noted in Table 2. Based on their colony morphology, mycelial growth, pigmentation and microscopic observations from a total of 12,545 cultures, 2584 were *F. oxysporium,* 2465*F. solani,* 1953 *Aspergillus* spp., 1703 *Penicillium* spp., 1294 *Botrytis* spp., 1278 *R. solani* and 1268 *Alternaria* spp*.* The results showed the morphology and mycelium characteristics of isolated strains, which will aid in the identification of isolated fungal strains in the future.

**Table 2:** Cultural and morphological characteristics of fungal isolates.

ReIsolates	Cultural characteristics		Morphological characteristics			Suggested genus	Reference
	Mycelial growth	Pigmentation	Septation	Conidia	Chlamydo spore		
GF_1_	Fluffy	White	Present	Macro conidia	Present	*F. oxysporum*	Leslie and Summerell, 2006
JF_5_	Fluffy	White	Present	Macro conidia	Both	*F. oxysporum*	
YF_3_	Fluffy	White	Present	Macro conidia	Present	*F. oxysporum*	
KF_2_	Fluffy	White	Present	Macro conidia	Present	*F. oxysporum*	
DF_4_	Fluffy	White	Present	Macro conidia	Present	*F. oxysporum*	
GF_3_	Velvet	Read	Present	Macro conidia	Absent	*F. solani*	Leslie and Summerell, 2006
JF_7_	Velvet	Light pinkish	Present	Both	Absent	*F. solani*	
YF_5_	Fluffy	Read	Present	Both	Present	*F. solani*	
KF_8_	Velvet	Grey purple	Present	Macro conidia	Present	*F. solani*	
DF_6_	Fluffy	Read	Present	Both	Absent	*F. solani*	
GF_8_	Appressed	Black	Absent	–	–	*R. solani*	Watanabe, 2002; Srinivas, 2016
JF_9_	Fluffy	Dark brown	Absent	–	–	*R. solani*	
YF_8_	Appressed	Black	Present	–	–	*R. solani*	
KF_10_	Fluffy	Dark brown	Absent	–	–	*R. solani*	
DF_9_	Fluffy	Dark brown	Absent	–	–	*R. solani*	

The frequency of occurrence of fungi ranged from 10.11 to 20.60% (Table 3). Out of 12,545 fungal colonies isolated from the faba bean seed samples, 20.60% were *F. oxysporium,* 19.65% were *F. solani,* 15.57% were *Aspergillus* spp., 13.58% were *Penicillium* spp*.,* 10.31% were *Botrytis* spp*.*, 10.19% were *R. solani* and 10.11% were *Alternaria* spp. (Table 3).

**Table 3:** Frequency occurrence of seedborne fungi on faba bean seed in the Ambo district.

Locations	*F. oxysporum*	*F. solani*	*Aspergillus* spp.	*Penicillium* spp.	*Botrytis* spp.	*R. solani*	*Alternaria* spp*.*	Total infection (%)
Golja	3.74	3.56	1.67	1.49	2.22	1.63	2.11	16.43
Jijigu Weransa	3.92	2.61	2.38	2.85	3.83	1.28	3.14	20.02
Dase Akililo	3.95	4.18	3.76	3.11	1.26	2.57	1.36	20.19
Ya’i Cabo	4.10	4.05	3.75	3.17	1.93	2.35	1.83	21.18
Kure Gatira	4.89	5.25	4.00	2.95	1.08	2.36	1.66	22.18
Total	20.60	19.65	15.57	13.58	10.31	10.19	10.11	100

### 3.3 Effects of seedborne fungi on the germination of seeds

The effect of the seedborne fungi on the germination of seed samples was determined after 7 days of incubation. The results from the germination percentage of seed samples revealed that the germination percentage ranged from 81% to 97%. A significant difference in germination percentage among the seed samples was observed. The seed samples obtained from Golja kebele showed the highest percentage of germination (97%). The germination percentage of seeds differed significantly from location to location and from farmer to farmer. Seed germination of a major faba bean-producing farmer’s association, Golja, was significantly higher (97%), followed by Jijigu Weransa (89%), but Kure Gatira had the lowest germination percentage (81%) (Table 4).

**Table 4:** Effects of seed-borne fungi on the germination of faba bean seeds.

Locations	Seed-borne fungi isolated							Total infection	Germination (%)
	*F. oxysporum*	*F. solani*	*Aspergillus* spp.	*Penicillium* spp.	*Botrytis* spp.	*R. solani*	*Alternaria* spp.		
Golja	469	447	210	167	304	205	263	2065	97.00
Jijigu Weransa	492	328	299	377	455	161	397	2509	89.00
Dase Akililo	501	566	489	374	141	299	165	2535	86.00
Ya’i Cabo	514	520	458	398	242	295	229	2656	85.00
Kure Gatira	608	604	497	387	152	318	214	2780	81.00
Total	2584	2465	1953	1703	1294	1278	1268	12,545	

Seed collected from Golja kebele in the farmer of seed sample code GF_2_ showed the highest germination (97%), followed by the seed sample code GF_5_, GF_1_ and JF_7_ with germination (96%, 94% and 93%), respectively, but the seed collected from Kure Gatira kebele in the farmer of seed sample code KF_8_ showed the lowest germination (81%), followed by the seed sample code YF_6_, DF_8_ and KF_7_ with germination (82%, 82% and 83%), respectively. The results also revealed that the sample with the highest fungal prevalence resulted in the lowest germination (Table 4).

Among the seedborne fungi transmitted from seed to seedlings, distinct symptoms of damping off, seed rot and seedling blight were observed for *Fusarium* spp. and *R. solani.* All transmitted seedborne fungi may serve as the primary source of infection to the faba bean crop. These fungi serve as the primary inoculum for the spread of diseases and have epidemiological significance. The widespread distribution of *Fusarium, Aspergillus* and *Penicillium* species may be attributed to the wide occurrence of these fungi on a wide range of substrates and their efficient spore dispersal mechanism. In the present study, *F. oxysporium* and *F. solani* were isolated from almost all seed samples with high frequency, but *R. solani* rarely occurred (Table 5). As observed from the results, three of the pathogens isolated from seed samples were *F. oxysporium, F. solani* and *R. solani*, which commonly cause root rot and damping-off disease of faba bean and are transmitted from seeds to seedlings in this experiment.

**Table 5:** List of fungi isolated from 50 faba bean seed samples by the agar plate method.

Locations	Sample code	Seed infection							Seedling infection			TES		
		FO	FS	ASS	PS	BS	RSO	ALS	FO	FS	RSO	FO	FS	RSO
Golja	GF_1_	62	23	20	0	0	16	38	21	0	11	33.87	0.00	68.75
	GF_2_	14	16	29	0	51	15	0	0	0	0	0.00	0.00	0.00
	GF_3_	56	50	0	29	15	35	20	16	17	23	28.57	34.00	65.71
	GF_4_	68	20	0	46	0	20	58	0	0	0	0.00	0.00	0.00
	GF_5_	43	50	60	0	0	0	0	0	20	0	0.00	40.00	0.00
	GF_6_	62	61	32	0	20	0	0	25	21	0	40.32	34.43	0.00
	GF_7_	42	52	20	53	58	0	50	20	12	0	47.62	23.08	0.00
	GF_8_	43	62	21	0	57	49	27	0	24	24	0.00	38.71	48.98
	GF_9_	17	60	27	0	60	23	20	0	0	0	0.00	0.00	0.00
	GF_10_	62	53	0	39	43	38	52	32	0	21	51.61	0.00	55.26
Jijigu Weransa	JF_1_	48	40	0	30	58	60	48	18	20	20	37.50	50.00	33.33
	JF_2_	55	26	0	52	60	0	0	25	0	0	45.45	0.00	0.00
	JF_3_	39	26	40	0	38	61	54	0	0	31	0.00	0.00	50.82
	JF_4_	52	18	38	46	51	0	0	15	0	0	28.85	0.00	0.00
	JF_5_	45	20	30	29	30	0	20	24	10	0	53.33	50.00	0.00
	JF_6_	62	39	30	45	59	0	67	22	9	0	35.48	23.08	0.00
	JF_7_	67	30	30	20	0	0	26	11	13	0	16.42	43.33	0.00
	JF_8_	38	32	60	60	60	0	50	0	13	0	0.00	40.63	0.00
	JF_9_	32	44	71	40	51	40	62	0	24	14	0.00	54.55	35.00
	JF_10_	54	53	0	55	53	0	67	14	15	0	25.93	28.30	0.00
Ya’i Cabo	YF_1_	72	45	39	60	30	0	39	23	19	0	31.94	42.22	0.00
	YF_2_	55	55	45	30	44	0	0	0	26	0	0.00	47.27	0.00
	YF_3_	46	57	46	0	0	66	0	20	22	21	43.48	38.60	31.82
	YF_4_	52	60	30	51	0	0	0	16	16	0	30.77	26.67	0.00
	YF_5_	24	52	56	30	57	0	58	0	0	0	0.00	0.00	0.00
	YF_6_	52	45	62	68	10	20	58	15	0	0	28.85	0.00	0.00
	YF_7_	72	56	0	75	0	63	20	0	16	33	0.00	28.57	52.38
	YF_8_	42	46	38	0	65	58	54	27	14	18	64.29	30.43	31.03
	YF_9_	30	52	68	36	0	20	0	20	18	0	66.67	34.62	0.00
	YF_10_	69	52	74	48	36	68	0	0	0	20	0.00	0.00	29.41
Kure Gatira	KF_1_	72	60	74	60	0	0	70	32	32	0	44.44	53.33	0.00
	KF_2_	31	51	57	0	0	56	0	11	21	16	35.48	41.18	28.57
	KF_3_	74	68	0	56	0	0	0	24	18	0	32.43	26.47	0.00
	KF_4_	73	60	50	63	16	58	0	23	10	18	31.51	16.67	31.03
	KF_5_	72	63	74	0	68	0	0	22	33	0	30.56	52.38	0.00
	KF_6_	55	70	72	53	0	30	0	19	17	0	34.55	24.29	0.00
	KF_7_	56	72	55	44	0	50	29	27	20	20	48.21	27.78	40.00
	KF_8_	76	59	39	48	29	59	43	26	0	21	34.21	0.00	35.59
	KF_9_	52	45	56	20	39	0	72	20	0	0	38.46	0.00	0.00
	KF_10_	47	56	20	43	0	65	0	0	0	23	0.00	0.00	35.38
Dase Akilio	DF_1_	45	71	62	68	19	16	0	0	21	0	0.00	29.58	0.00
	DF_2_	49	61	42	0	0	50	0	19	16	15	38.78	26.23	30.00
	DF3	40	46	49	57	0	30	49	20	23	0	50.00	50.00	0.00
	DF_4_	48	31	60	30	60	39	0	18	0	19	37.50	0.00	48.72
	DF_5_	65	48	30	47	0	40	0	26	18	24	40.00	37.50	60.00
	DF_6_	47	65	48	10	62	0	58	0	17	0	0.00	26.15	0.00
	DF_7_	64	65	55	10	0	0	0	16	25	0	25.00	38.46	0.00
	DF_8_	51	70	46	70	0	14	58	21	28	0	41.18	40.00	0.00
	DF_9_	50	59	30	22	0	37	0	10	0	18	20.00	0.00	48.65
	DF_10_	42	50	67	59	0	74	0	0	0	27	0.00	0.00	36.49

Where FO - *Fusarium oxysporum*, FS - *Fusarium solani*, ASS - *Aspergillus* species, PS - *Penicillium* species, BS - *Botrytis* species, ROS - *Rihzoctoniasolani*, ALS - *Alternaria* species and TES - Transmission efficiency.

### 3.4 Antifungal activities of plant extracts

The in vitro antifungal effects of seven plants extracted with hot and cold water against the mycelial growth of *F. oxysporium*, *F. solani* and *R. solani* of test fungi by the poisoned food technique are shown as follows. The results presented in Table 6 revealed that all plant extracts significantly inhibited the mycelial growth of the three tested fungi (p < 0.05) at all tested concentrations and both types of aqueous extracts. However, the effects varied with the concentration of the extracts. The mycelial growth of the test fungi decreased as the concentration percentage of plants increased in both hot and cold water extracts, but when comparing the hot water extract with the cold water extract, the greatest inhibitory effect was shown by the hot water extract compared with the cold water extract, as shown in Table 6. Based on this, the impacts of various treatments on the mycelium growth of tested fungi in comparison with control/untreated plate the mycelium growth of the tested fungi has been influenced differently by the plant extracts in comparison to each other and test fungal pathogens (Supplementary material 1, Table S1–7).

**Table 6:** Antagonistic effect of different plant extracts on mycelial growth and inhibition zone percentage of the three test pathogens isolated from faba bean seed samples.

Extraction method	Concentration (%)	*Fusarium oxysporium*		*Fusarium solani*		*Rhizoctoni solani*	
		Mycelia growth (mm)*	Inhibition zone (%)	Mycelia growth (mm)*	Inhibition zone (%)	Mycelia growth (mm)*	Inhibition zone (%)
Cold	0	40.75^a^	0.00^g^	37.76^a^	0.00^g^	40.25^a^	0.00^g^
	5	17.00^b^	57.70^f^	16.31^b^	56.03^f^	17.26^b^	56.41^f^
	10	12.83^d^	67.97^d^	12.33^d^	66.72^d^	13.17^d^	66.73^d^
	20	8.69^f^	78.28^b^	8.50^f^	77.02^b^	8.98^f^	77.28^b^
Hot	0	40.25^a^	0.00^g^	37.25^a^	0.00^g^	39.75^a^	0.00^g^
	5	15.83^c^	60.10^e^	15.33^c^	59.23^e^	16.17^c^	59.66^e^
	10	11.67^e^	71.22^c^	11.17^e^	70.27^c^	12.00^e^	70.05^c^
	20	7.52^g^	81.42^a^	7.33^g^	80.45^a^	7.83^g^	80.42^a^
CV (%)		3.94	3.45	3.47	3.19	3.02	2.71
LSD (0.05)		0.44	1.03	0.36	0.94	0.34	0.8

**Note:** *Mean values are the mean of three replicates. Means followed by the same letter are not significantly different according to Duncan’s multiple ranges (P < 0.05), CV = coefficient of variation, and LSD = least significant difference.

The antagonistic effects of different plant extracts at different concentrations (5%, 10% and 20%) on mycelial growth and the inhibition zone of the tested fungi were significantly (P ≤ 0.01) different between the treatments. Among the plant extracts at 20% concentration, *A. sativum* L. caused the lowest mycelial growth of *F. solani, F. oxysporum and R. solani* (6.57 mm, 6.58 mm and 6.92 mm, respectively), followed by *C. longa* L. (7.00 mm, 7.08 mm and 7.42 mm, respectively) and *P. dodecandra* (7.42 mm, 7.58 mm and 7.92 mm, respectively), whereas the highest mycelial growth was recorded at the same concentrations extract of *N. physalodes* (L.) Gaertn. (9.42 mm, 9.58 mm and 9.83 mm, respectively). At a 5% concentration, the mycelial growth of the three test pathogens was recorded above 14.42 mm in all plant extract tests. Based on their mycelial growth, the inhibition zone percentages of *F. oxysporum, F. solani* and *R.* solani corresponding to seven plant extracts at 5%, 10% and 20% concentrations.

For both extraction methods (5%, 10% and 20%), the concentration revealed significant mycelial growth and inhibition zone percentage compared to each other (Table 6). Among them, at 20% concentration, the plants extracted in hot water caused the lowest mycelial growth of *F. solani, F. oxysporum and R. solani* (7.33 mm, 7.52 mm and 7.83 mm, respectively), followed by 10% concentration (11.17 mm, 11.67 mm and 12.00 mm, respectively), whereas the highest mycelial growth was recorded at the same extraction method of 5% concentration (15.33 mm, 15.83 mm and 16.17 mm, respectively). Based on their mycelial growth, the inhibition zone percentages of *F. oxysporum, F. solani* and *R.* solani affected at 5%, 10% and 20% concentrations of both extraction methods are shown in Table 6.

### 3.5 Evaluation of bioagents on mycelial growth of selected fungi under in vitro conditions

**Fig. 2:**
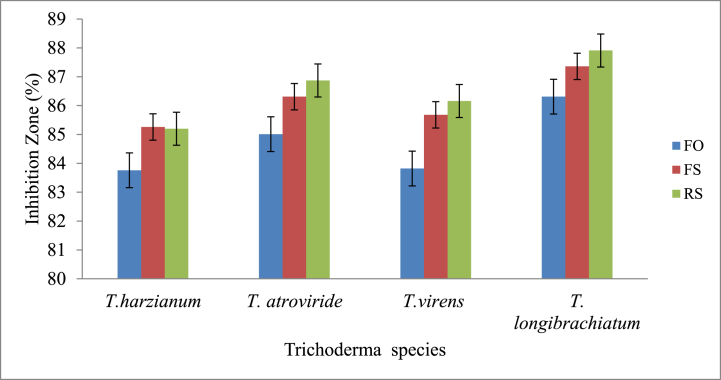
Effects of different *Trichoderma* species on the inhibition zone percentage of the three test pathogens (FO is *F. oxsporium,* FS is *F. solani* and RS is *R. solani*).

The inhibitory effects of *T. harzianum, T. longibrachiatum, T. atroviride,* and *Trichoderma virens* against the mycelial growth of the three test fungi *F. oxysporum, F. solani* and *R. solani* in the dual culture method are presented in Fig. 2 and Supplementary material 1, Table S8–13. The antagonistic effects of *Trichoderma* spp. against the mycelia growth of *F. oxysporum* were in the range of 5.50 mm–6.53 mm. *T. longibrachiatum* showed the highest inhibitory effect on mycelial growth (5.50 mm), followed by *T. atroviride* (6.00 mm), *Trichoderma virens* (6.50 mm) and *T. harzianum* (6.53 mm).

These results showed that the growth inhibition of *F. solani* by *Trichoderma* spp. was in the range of 5.00 mm–5.83 mm. *T. longibrachiatum* also inhibited the mycelial growth of *F. solani*, where the growth inhibition was 5.00 mm, followed by *T. atroviride* (5.42 mm), *Trichoderma virens* (5.67 mm) and *T. harzianum* (5.83 mm). The current study showed that the mycelial growth of *R. solani* by *Trichoderma* spp. was in the range of 4.83 mm–5.82 mm. *T. longibrachiatum* inhibited the mycelial growth of *R. solani* by 4.83 mm, followed by *T. atroviride* (5.25 mm), *Trichoderma virens* (5.53 mm) and *T. harzianum* (5.82 mm). The highest growth inhibition against the three isolated test fungi (*R. solani, F. solani* and *F. oxysporum*) was recorded by *T. longibrachiatum* (4.83 mm, 5.00 mm and 5.50 mm), while the lowest effect was recorded by *T. harzianum* (5.82 mm, 5.83 mm and 6.53 mm). Based on their mycelial growth, the inhibition zone percentages of *F. oxysporum, F. solani* and *R. solani* affected by *Trichoderma* species (*T. harzianum, T. longibrachiatum, T. atroviride,* and *T. virens)* are shown in Fig. 2.

## 4 Discussion

According to experimental work on the seedborne fungi associated with faba bean seeds, seven species of fungi were isolated and identified. Faba bean is susceptible to several fungal diseases that decrease production and lower the quality of seeds. The results of the study revealed that *F. oxysporum*, *F. solani*, *Aspergillus* spp. *Penicillium* spp. *Botrytis* spp. *R. solani* and *Alternaria* spp. were observed and identified. These results are in agreement with the findings of other researchers [15,[38], [39], [40], [41]]. Among the isolated fungi, *Fusarium* and *Aspergillus* species were the most predominant fungi in all seed samples. Seed-to-seedling transmission tests indicated that *F. oxysporum, F. solani* and *R. solani* isolates were the most common fungal isolates that significantly induced damping-off and root rot disease on faba bean plants. This result is similar to the findings of Elliot and Crowford [42], who found these fungal pathogenic organisms to be the most devastating rot-causing organisms in bean crops in different locations in the world [43]. These results showed that several root rot and wilt pathogens, such as *F. oxysporum, R. solani* and *F. solani,* are reported to attack faba bean roots and stem bases, causing serious losses in seed germination and seedlings. These results agree with those recorded by Abdel-Kader et al. [44]. Seedborne pathogen invasion reduces germination and nutrition and is also responsible for producing mycotoxins and loss of quality [45]. Seedborne diseases serve as primary inocula for the infection of the next developing crops, thereby reducing yields and qualities of the produce and playing a role in spreading the diseases to new areas [44]. The seedborne pathogens associated with seeds externally or internally may cause various infections, such as seed necrosis, reduction or elimination of germination capacity, and seedling damage, resulting in the development of disease at later stages of plant growth by systemic infection [46]. Infected seeds play a key role in the dissemination of plant pathogens and disease establishment [47]. The results of these studies are similar to those obtained by earlier workers and show that *Fusarium* is the most dominant species isolated from maize [[48], [49], [50]], and in Egypt, the main pathogens responsible for damping-off and wilt incidence of beans are *R. solani* (Kühn) and *F. oxysporum* f. sp. phaseoli [51].

Biological control of the three isolated test fungi by seven plant extracts and four *Trichoderma* spp. have been evaluated. The results revealed that all the tested plant extracts at 5%, 10% and 20% concentrations significantly inhibited the mycelial growth of all test fungi, and the four *Trichoderma* species also exhibited the strongest antagonistic activity. The inhibitory action of the aqueous plant extracts on mycelial growth increased with an increase in concentrations, and the hot water extracts provided higher effects/toxicity than the cold water extract in all test fungi. The results highlight the highest inhibitory effect of *A. sativum* L. extracted at 20% concentration against mycelial growth inhibition of the three test fungi (*F. oxysporum, R. solani* and *F. solani*), but *N. physalodes* (L.) Gaertn. extract at the same concentration showed the lowest inhibitory effects on the three test fungi. These results revealed that the antifungal activities of the extracts were enhanced by increasing the concentration from 5 to 20% (w/v); hence, the inhibition activities of the extracts were concentration dependent. This is in agreement with the reports of Ilondu [52], Chiejina and Ukeh [53], and Jasso et al. [54], who indicated that an increase in antifungal activities corresponded to an increase in the concentration of plant extracts.

The antagonistic effects of *Trichoderma* spp. against the three test fungi also indicated significant inhibitory activities. Mycelial growth inhibition percentages of all *Trichoderma* spp. were recorded above 50% in the three test pathogens. The best inhibitory effects against *R. solani*, *F. solani* and *F. oxysporum* were obtained by *T. longibrachiatum* (87.91%, 87.36% and 86.31%), followed by *T. atroviride* (86.87%, 86.31% and 85.01%), *Trichoderma virens* (86.16%, 85.68% and 83.82%) and *T. harzianum* (85.45%, 85.26%, and 83.74%), respectively. These results agree with those recorded by El-Kafrawy [55] and Lewis et al. [56]. The antagonistic activity of *Trichoderma* spp. against *Fusarium* spp*.* also coincides with the result reported by Morsy et al. [57]. The traditional medicinal and other uses of plants extracted in this study, i.e., *M. senegalensis* Loes., *A. vera* (L.) Burm.f, *N. physalodes* (L.) Gaertn., *Nicotiana tabacum* L., *Curcuma longa* L., and *P. dodecandra* L’Hér. have been reported in Ethiopia [[58], [59], [60], [61]].

The traditional medicinal use of plants extracted in this study, i.e., Kombolcha leaf (*Maytenus senegalensis* Loes.), Argissa (*Aloe vera* (L.) Burm.f), Abshoo leaf (*N. physalodes* (L.) Gaertn.), tabaco leaf (*Nicotiana tabacum* L.), Turmeric rhzome (*Curcuma longa* L.) and endod seeds (*Phytolacca dodecandra* L’Hér.) has been reported in Ethiopia [[58], [59], [60], [61]]. In vivo and in vitro studies have shown that *A. vera* species has several biological activities, such as antifungal, antioxidant and antiviral properties [62]. Due to the restrictive application of fungicides, natural alternatives to synthetic fungicides, such as the use of essential oils, have shown efficacy in reducing decay [63]. The jel of *A. vera* could be applied as a treatment before harvesting to inhibit fungal spoilage and reduce the incidence of decay after harvesting storage of table grapes [64]. Aqueous extract of *A. sativum* has been used to treat plantlets of *Harpagophytum procumbens* by presoaking plantlets in the extract prior to planting out into the pot or by applying the extract as a soil drench after planting plantlets into the pot [65]. In a field trial, fungal disease severity reduction was achieved by the extraction of *M. senegalensis* Loes at a 5% concentration against *Phytophthora infestans* [66].

Alkaloid mixture, petroleum ether, and withanicandrin of *N. physaloides* Gaertn showed antifungal activities against *S. cerevisiae* and *C.* albicans [67]. Different compounds isolated from *N. tabacum*, such as alkaloids, nicotine, and capsidiol, have higher inhibitory activity against the fungus *Phytophthora nicotianae* [68]. *C. longa* has been reported to have toxic activity on fungi involved in the deterioration of crops by interfering with the development of mycelia [69]. It has been reported that the ethanol and hexane extracts of *C. longa* have significant antifungal activity against pathogenic fungi, such as *Botrytis cinerea, Chaetomium olivaceum, Fusarium graminearum,* and *Mycogone perniciosa* [70,71]. The methanol extract of its root had an antifungal effect against *Candida albicans*, *Cryptococcus neoformans, Microsporum gypseum*, and *Trichophyton mentagrophytes* [72]. Organic compound emitted from *Trichoderma* spp. inhibit mycelial growth of *P. infestans* grown on laboratory media and on potato tubers [73].

## 5 Conclusions and recommendations

The results obtained from this study showed that the extracts of different screened plants exhibit antifungal effects against *F. oxysporum, F.* solani and *R. solani.* In particular, hot water extracts of *A. sativum* L.*, M. senegalensis* Loes., *A. vera* (L.) Burm.f*, N. physalodes* (L.) Gaertn., *N. tabacum* L., *C. longa* L. and *P. dodecandra* L’Hér. offer effective bioactive compounds for mycelial growth of the test fungi. The tested *Trichoderma* spp. also showed a good antagonistic effect against the three test fungi. These results indicate that plant extracts and *Trachoderma* spp. can be a potential alternative management option against plant disease and can be used as a seed treatment or seed coating. Hot water extracts of tested plants were revealed to be relatively more effective against the pathogen than cold water extracts. Active compounds of all test plants are extractable with water, and most are extracted with hot water. The results of this study revealed that cold and hot aqueous extracts of all the plants possess diverse antifungal activity against the test fungi. The differentiating activities against the selected isolate of these plant extracts encourage the development of broad-spectrum antifungals in the future. All the tested plant extracts and *Trichoderma* spp. contain antifungal agents and can be used against seedborne fungi in faba bean but need further purification for better efficacy. The results also confirmed that continuous effort to search for excellent plant extracts and antagonistic microorganisms is primarily needed. The results of the present study can be further exploited and tested under greenhouse and field experiments to formulate an integrated disease management schedule for root rot and damping-off disease of faba bean.

## Author contribution statement

Amsalu Neme, Ararsa Leta, Amin Mohammed Yones: Conceived and designed the experiments; performed the experiments; analysed and interpreted the data and wrote the paper.

Muhidin Tahir: Analysed and interpreted the data and wrote the paper.

## Data availability statement

Data will be made available on request.

## Additional information

Supplementary content related to this article has been published online at [URL].

## Declaration of competing interest

The authors declare that they have no known competing financial interests or personal relationships that could have appeared to influence the work reported in this paper.

## Appendix A Supplementary data

suplimantary material-R3 suplimantary material-R3
