# Supplementary material for: Seedborne mycoflora of faba bean (Vicia fabae L.) and evaluation of plant extract and Trichoderma species against mycelium growth of selected fungi
Source: Heliyon. 2023 Jun 15;9(6):e17291. doi: 10.1016/j.heliyon.2023.e17291 (PMC10293731; doi:10.1016/j.heliyon.2023.e17291)
Supplement: suplimantary material-R3 [file mmc1.docx]

**Supplementary material 1:** Antagonistic effect of different plant extracts and *Trichoderma* species on mycelial growth and figures of the appearance of fungal pathogens on faba bean seeds.

**Supplementary material 1, Table S1** Antagonistic effect of different plant extracts on mycelial growth and inhibition zone percentage of the three test pathogens isolated from faba bean seed

| Scientific name | Concentration (%) | *Fusarium oxysporium* | | *Fusarium solani* | | *Rhizoctonia solani* | |
| --- | --- | --- | --- | --- | --- | --- | --- |
|  |  | Mycelia Growth (mm)* | Inhibition Zone  (%) | Mycelia Growth (mm)* | Inhibition Zone  (%) | Mycelia Growth (mm)* | Inhibition Zone  (%) |
| *Allium sativum* L. | 0 | 42.75^a^ | 0.00^s^ | 39.75^a^ | 0.00^s^ | 42.25^a^ | 0.00^s^ |
|  | 5 | 14.92^k^ | 64.03^lm^ | 14.42^k^ | 62.39^lm^ | 15.25^l^ | 62.68^lm^ |
|  | 10 | 10.75^p^ | 74.83^fg^ | 10.25^qr^ | 73.24^fg^ | 11.08^r^ | 73.76^fg^ |
|  | 20 | 6.58^t^ | 84.60^a^ | 6.57^x^ | 83.47^a^ | 6.92^x^ | 83.61^a^ |
| *Aloe vera* (L.) Burm.f | 0 | 40.50^cd^ | 0.00^s^ | 37.50^cd^ | 0.00^s^ | 40.00^cd^ | 0.00^s^ |
|  | 5 | 16.42^hij^ | 59.45^op^ | 15.92^hi^ | 57.53^op^ | 16.75^ij^ | 58.10^op^ |
|  | 10 | 12.25^mno^ | 69.73^ij^ | 11.75^no^ | 68.64^ij^ | 12.58^op^ | 68.54^ij^ |
|  | 20 | 8.25^rs^ | 79.63^cd^ | 7.92^uv^ | 78.87^cd^ | 8.42^uv^ | 78.94^cd^ |
| *Curcuma longa* L. | 0 | 42.00^ab^ | 0.00^s^ | 38.92^ab^ | 0.00^s^ | 41.50^ab^ | 0.00^s^ |
|  | 5 | 15.42^jk^ | 63.29^mn^ | 14.92^jk^ | 61.66^mn^ | 15.75^kl^ | 62.03^mn^ |
|  | 10 | 11.25^op^ | 73.18^gh^ | 10.75^pq^ | 72.36^gh^ | 11.58^qr^ | 72.07^gh^ |
|  | 20 | 7.08^t^ | 83.10^ab^ | 7.00^wx^ | 82.01^ab^ | 7.42^wx^ | 82.10^ab^ |
| *Maytenus senegalensi* Loes. | 0 | 39.00^ef^ | 0.00^s^ | 36.13^ef^ | 0.00^s^ | 38.50^ef^ | 0.00^s^ |
|  | 5 | 17.42^gh^ | 55.33^qr^ | 16.75^gh^ | 53.55^qr^ | 17.67^gh^ | 54.03^qr^ |
|  | 10 | 13.25^lm^ | 66.00^kl^ | 12.75^lm^ | 64.69^kl^ | 13.58^mn^ | 64.69^kl^ |
|  | 20 | 9.08^qr^ | 76.69^ef^ | 8.92^st^ | 75.28^ef^ | 9.42^st^ | 75.52^ef^ |
| *Nicandra physalodes* (L.) Gaertn. | 0 | 38.25^f^ | 0.00^s^ | 35.25^f^ | 0.00^s^ | 37.75^f^ | 0.00^s^ |
|  | 5 | 17.92^g^ | 53.14^r^ | 17.08^g^ | 51.51^r^ | 18.17^g^ | 51.85^r^ |
|  | 10 | 13.75^l^ | 65.41^klm^ | 13.25^l^ | 63.72^lm^ | 14.08^m^ | 63.89^lm^ |
|  | 20 | 9.58^q^ | 74.94^fg^ | 9.42^rs^ | 73.24^fg^ | 9.83^s^ | 73.94^fg^ |
| *Nicotiana tabacum* L. | 0 | 39.75^de^ | 0.00^s^ | 36.75^de^ | 0.00^s^ | 39.25^de^ | 0.00^s^ |
|  | 5 | 16.92^ghi^ | 57.43^pq^ | 16.25^ghi^ | 55.77^pq^ | 17.17^hi^ | 56.25^pq^ |
|  | 10 | 12.75^lmn^ | 67.92^jk^ | 12.25^mn^ | 66.64^jk^ | 13.08^no^ | 66.66^jk^ |
|  | 20 | 8.58^qrs^ | 78.38^de^ | 8.42^tu^ | 77.08^de^ | 8.92^tu^ | 77.26^de^ |
| *Phytolacca dodecandra* L’Hér. | 0 | 41.25^bc^ | 0.00^s^ | 38.25^bc^ | 0.00^s^ | 40.75^bc^ | 0.00^s^ |
|  | 5 | 15.92^ijk^ | 61.39^no^ | 15.42^ij^ | 59.66^no^ | 16.25^jk^ | 60.10^no^ |
|  | 10 | 11.75^nop^ | 71.50^hi^ | 11.25^op^ | 70.57^hi^ | 12.08^pq^ | 70.33^hi^ |
|  | 20 | 7.58^st^ | 81.61^bc^ | 7.42^vw^ | 80.58^bc^ | 7.92^vw^ | 80.59^bc^ |
| Cv (%) | | 3.94 | 3.45 | 3.47 | 3.19 | 3.02 | 2.71 |
| LSD (0.05) | | 0.44 | 1.03 | 0.36 | 0.94 | 0.34 | 0.8 |

**Note:** Mean values are the mean of three replicates. Means followed by the same letter are not significantly different according to Duncan’s multiple ranges (P < 0.05). CV=coefficient of variation and LSD= least significant difference.

**Supplementary material, Figure 1** Plate A shows apparently healthy seeds, and plate B shows the appearance of fungal pathogens on faba bean seeds.

**Supplementary material, Figure 2** Damping off and root rot symptoms from the 7-day plate method test of faba bean seeds

Supplementary material 1, Table S2: Effects of different plant extracts on the mycelial growth of *F.* *oxysporum*

| Source | DF | Sum of square | Mean square | F value | Pr > F |
| --- | --- | --- | --- | --- | --- |
| Model | 57 | 26841.50744 | 470.90364 | 811.52 | <.0001* |
| Rep | 2 | 0.50298 | 0.25149 | 0.43 | 0.649* |
| BT | 6 | 23.66071 | 3.94345 | 6.8 | <.0001* |
| ExM | 1 | 23.625 | 23.625 | 40.71 | <.0001* |
| CN | 3 | 26574.86161 | 8858.2872 | 15266 | <.0001* |
| BT*ExM | 6 | 0 | 0 | 0 | 1 |
| BT*CN | 18 | 196.98214 | 10.94345 | 18.86 | <.0001* |
| ExM*CN | 3 | 21.875 | 7.29167 | 12.57 | <.0001* |
| BT*ExM*CN | 18 | 0 | 0 | 0 | 1 |
| Error | 110 | 63.83036 | 0.58028 |  |  |
| Corrected Total | 167 | 26905.3378 |  |  |  |

Note: Statistical tests were performed using analysis of variance (ANOVA). shows a significant difference at (*p* < 0.05) between averages of the paired categories

Supplementary material 1, Table S3: The effects of different plant extracts on the inhibition zone percentage of *F.* *oxysporum*

| Source | DF | Sum of square | Mean square | F value | Pr > F |
| --- | --- | --- | --- | --- | --- |
| Model | 57 | 163418.9891 | 2866.9998 | 886.03 | <.0001 |
| Rep | 2 | 4.0497 | 2.0249 | 0.63 | 0.537 |
| BT | 6 | 1240.6319 | 206.772 | 63.9 | <.0001 |
| ExM | 1 | 246.6226 | 246.6226 | 76.22 | <.0001 |
| CN | 3 | 161414.5122 | 53804.8374 | 16628 | <.0001 |
| BT*ExM | 6 | 1.1355 | 0.1893 | 0.06 | 0.999 |
| BT*CN | 18 | 428.884 | 23.8269 | 7.36 | <.0001 |
| ExM*CN | 3 | 82.3521 | 27.4507 | 8.48 | <.0001 |
| BT*ExM*CN | 18 | 0.8012 | 0.0445 | 0.01 | 1 |
| Error | 110 | 355.9347 | 3.2358 |  |  |
| Corrected Total | 167 | 163774.9238 |  |  |  |

Notice: - BT=Botanicals, CN=Concentration, ExM=Extraction Method, Rep=Replication

Supplementary material 1, Table S4: The effects of different plant extracts on the mycelial growth of *F. solani*

| Source | DF | Sum of square | Mean square | F value | Pr > F |
| --- | --- | --- | --- | --- | --- |
| Model | 57 | 22331.14472 | 391.77447 | 978.49 | <.0001 |
| Rep | 2 | 0.41592 | 0.20796 | 0.52 | 0.596 |
| PT | 6 | 21.32515 | 3.55419 | 8.88 | <.0001 |
| ExM | 1 | 20.54501 | 20.54501 | 51.31 | <.0001 |
| CN | 3 | 22080.13207 | 7360.04402 | 18382 | <.0001 |
| BT*ExM | 6 | 0.23884 | 0.03981 | 0.1 | 0.996 |
| BT*CN | 18 | 187.17783 | 10.39877 | 25.97 | <.0001 |
| ExM*CN | 3 | 20.79576 | 6.93192 | 17.31 | <.0001 |
| BT*ExM*CN | 18 | 0.51414 | 0.02856 | 0.07 | 1 |
| Error | 110 | 63.83036 | 0.58028 |  |  |
| Corrected Total | 167 | 26905.3378 |  |  |  |

Supplementary material 1, Table S5: The effects of different plant extracts on the inhibition zone percentage of *F. solani*

| Source | DF | Sum of square | Mean square | F value | Pr > F |
| --- | --- | --- | --- | --- | --- |
| Model | 57 | 158475.6642 | 2780.2748 | 1039 | <.0001 |
| Rep | 2 | 0.41592 | 0.20796 | 0.52 | 0.596 |
| PE | 6 | 21.32515 | 3.55419 | 8.88 | <.0001 |
| ExM | 1 | 20.54501 | 20.54501 | 51.31 | <.0001 |
| CN | 3 | 22080.13207 | 7360.04402 | 18382 | <.0001 |
| BT*ExM | 6 | 0.23884 | 0.03981 | 0.1 | 0.996 |
| BT*CN | 18 | 187.17783 | 10.39877 | 25.97 | <.0001 |
| ExM*CN | 3 | 20.79576 | 6.93192 | 17.31 | <.0001 |
| BT*ExM*CN | 18 | 0.51414 | 0.02856 | 0.07 | 1 |
| Error | 110 | 63.83036 | 0.58028 |  |  |
| Corrected Total | 167 | 26905.3378 |  |  |  |

Note: - PT=Plant extract, CN=Concentration, ExM=Extraction method, Rep=Replication

Supplementary material 1, Table S6: The effects of different plant extracts on the mycelial growth of *R. solani*

| Source | DF | Sum of squares | Mean square | F value | Pr > F |
| --- | --- | --- | --- | --- | --- |
| Model | 57 | 25415.50744 | 445.8861 | 1292.9 | <.0001 |
| Rep | 2 | 0.89583 | 0.44792 | 1.3 | 0.277 |
| PE | 6 | 21.97619 | 3.6627 | 10.62 | <.0001 |
| ExM | 1 | 22.14881 | 22.14881 | 64.22 | <.0001 |
| CN | 3 | 25155.17113 | 8385.05704 | 24313 | <.0001 |
| BT*ExM | 6 | 0.03869 | 0.00645 | 0.02 | 1 |
| BT*CN | 18 | 194.13095 | 10.78505 | 31.27 | <.0001 |
| ExM*CN | 3 | 21.07738 | 7.02579 | 20.37 | <.0001 |
| BT*ExM*CN | 18 | 0.06845 | 0.0038 | 0.01 | 1 |
| Error | 110 | 37.9375 | 0.34489 |  |  |
| Corrected Total | 167 | 25453.44494 |  |  |  |

Supplementary material 1, Table S7 The effects of different plant extracts on the inhibition zone percentage of *R. solani*

| Source | DF | Sum of square | Mean square | F value | Pr > F |
| --- | --- | --- | --- | --- | --- |
| Model | 57 | 158608.4898 | 2782.6051 | 1437.9 | <.0001 |
| Rep | 2 | 4.3293 | 2.1646 | 1.12 | 0.33 |
| PE | 6 | 1257.4158 | 209.5693 | 108.29 | <.0001 |
| ExM | 1 | 247.1803 | 247.1803 | 127.73 | <.0001 |
| CN | 3 | 156585.0836 | 52195.0279 | 26971 | <.0001 |
| BT*ExM | 6 | 0.153 | 0.0255 | 0.01 | 1 |
| BT*CN | 18 | 431.2587 | 23.9588 | 12.38 | <.0001 |
| ExM*CN | 3 | 82.5641 | 27.5214 | 14.22 | <.0001 |
| BT*ExM*CN | 18 | 0.5049 | 0.0281 | 0.01 | 1 |
| Error | 110 | 212.8769 | 1.9352 |  |  |
| Corrected Total | 167 | 158821.3667 |  |  |  |

Note: - PE= plant extract, CN=concentration, ExM=extraction method, Rep=replication

Supplementary material 1, Table S8 The effects of different *Trichoderma* species on the mycelial growth of *F. oxysporum*

| Source | DF | Sum of square | Mean square | F value | Pr > F |
| --- | --- | --- | --- | --- | --- |
| Model | 6 | 2783.467893 | 463.911316 | 835.48 | <.0001 |
| Replication | 2 | 1.228853 | 0.614427 | 1.11 | 0.377 |
| Treatment | 4 | 2782.23904 | 695.55976 | 1252.7 | <.0001 |
| Error | 8 | 4.44208 | 0.55526 |  |  |
| Corrected Total | 14 | 2787.909973 |  |  |  |

Supplementary material 1, Table S9 The effects of different *Trichoderma* species on the inhibition zone percentage of *F. oxysporum*

| Source | DF | Sum of square | Mean square | F value | Pr > F |
| --- | --- | --- | --- | --- | --- |
| Model | 6 | 17245.2312 | 2874.2052 | 1281.3 | <.0001* |
| Replication | 2 | 4.53817 | 2.26909 | 1.01 | 0.406 |
| Treatment | 4 | 17240.69303 | 4310.17326 | 1921.5 | <.0001* |
| Error | 8 | 17.94549 | 2.24319 |  |  |
| Corrected Total | 14 | 17263.17669 |  |  |  |

Note: Statistical tests were performed using analysis of variance (ANOVA). shows a significant difference at (*p* < 0.05) between averages of the paired categories

Supplementary material 1, Table S10 The effects of different *Trichoderma* species on the mycelial growth of *F. solani*

| Source | DF | Sum of square | Mean square | F value | Pr > F |
| --- | --- | --- | --- | --- | --- |
| Model | 6 | 2792.933333 | 465.488889 | 1680 | <.0001 |
| Replication | 2 | 0.325 | 0.1625 | 0.59 | 0.579 |
| Treatment | 4 | 2792.608333 | 698.152083 | 2519.7 | <.0001 |
| Error | 8 | 2.216667 | 0.277083 |  |  |
| Corrected Total | 14 | 2795.15 |  |  |  |

Supplementary material 1, Table S11 The effects of different *Trichoderma* species on the inhibition zone percentage of *F. solani*

| Source | DF | Sum of square | Mean square | F value | Pr > F |
| --- | --- | --- | --- | --- | --- |
| Model | 6 | 2792.933333 | 465.488889 | 1680 | <.0001 |
| Replication | 2 | 1.74705 | 0.87353 | 0.62 | 0.562 |
| Treatment | 4 | 17820.58071 | 4455.14518 | 3166.6 | <.0001 |
| Error | 8 | 11.25521 | 1.4069 |  |  |
| Corrected Total | 14 | 17833.58297 |  |  |  |

Supplementary material 1, Table S12 The effects of different *Trichoderma* species on the mycelial growth of *R. solani*

| Source | DF | Sum of square | Mean square | F value | Pr > F |
| --- | --- | --- | --- | --- | --- |
| Model | 6 | 2792.933333 | 465.488889 | 1680 | <.0001 |
| Replication | 2 | 0.081333 | 0.040667 | 0.26 | 0.778 |
| Treatment | 4 | 2877.834333 | 719.458583 | 4572.8 | <.0001 |
| Error | 8 | 1.258667 | 0.157333 |  |  |
| Corrected Total | 14 | 2879.174333 |  |  |  |

Supplementary material 1, Table S13 The effects of different *Trichoderma* species on the inhibition zone percentage of *Rhizoctonia solani*

| Source | DF | Sum of squares | Mean square | F value | Pr > F |
| --- | --- | --- | --- | --- | --- |
| Model | 6 | 17985.60912 | 2997.60152 | 5024.7 | <.0001 |
| Replication | 2 | 1.18969 | 0.59485 | 1 | 0.411 |
| Treatment | 4 | 17984.41943 | 4496.10486 | 7536.6 | <.0001 |
| Error | 8 | 4.77257 | 0.59657 |  |  |
| Corrected Total | 14 | 17990.38169 |  |  |  |
